# Effect of Exercise Conditioning on Countering the Effects of Obesity and Insulin Resistance in Horses—A Review

**DOI:** 10.3390/ani14050727

**Published:** 2024-02-26

**Authors:** Shannon Pratt-Phillips

**Affiliations:** Department of Animal Science, North Carolina State University, Raleigh, NC 27607, USA; sepratt2@ncsu.edu; Tel.: +1-(919)-513-1117

**Keywords:** equine, obesity, exercise, insulin dysregulation

## Abstract

**Simple Summary:**

Obesity is a global concern within human, pet, and horse populations. A major consequence of obesity is a disruption to glucose and insulin metabolisms, leading to metabolic conditions including insulin resistance and/or diabetes. This paper discusses these consequences of obesity and excess adipose tissue both in idle and athletic subjects. Exercise conditioning can improve glucose metabolism and insulin sensitivity. Regular exercise can also facilitate weight loss, wherein the reduction of adipose tissue alleviates fat’s negative impacts on metabolism, while also decreasing the mechanical load on a subject’s limbs. These actions could result in better health, reduced incidence of metabolic disease, and, perhaps, improved performance.

**Abstract:**

Obesity is an important health concern in horses, along with humans and companion animals. Adipose tissue is an inflammatory organ that alters the insulin-signaling cascade, ultimately causing insulin dysregulation and impaired glucose metabolism. These disruptions can increase the risk of metabolic disease and laminitis in horses and may also impact energy metabolism during exercise. A single bout of exercise, along with chronic exercise conditioning, increases insulin sensitivity and glucose disposal via both contraction- and insulin-mediated glucose uptake pathways. Regular exercise also increases calorie expenditure, which can facilitate weight (as body fat) loss. This paper explores the metabolic pathways affected by adiposity, as well as discusses the impact of exercise on insulin metabolism in horses.

## 1. Introduction

It is well documented that exercise conditioning has profound impacts on health and wellbeing. Exercise is recognized by the American Medical Association as “one of the most important contributors to a healthy lifestyle” [1]. The American College of Sports Medicine launched the “Exercise is Medicine” program to highlight the importance of physical activity, particularly for the anti-inflammatory benefits of exercise [2]. Exercise conditioning results in improvements in performance capacity, cardio-respiratory health, musculature efficiency, biomechanics, and bone remodeling. A reduction in systemic inflammation is a key element of the benefits of exercise conditioning, which also has impacts on insulin and glucose metabolism. Exercise may also decrease body fat and increase muscle mass. Exercise is prescribed to mediate inflammation and obesity and to improve health, and yet exercise may be challenging to some. An obesity-induced decline in exercise capacity (OIDEC) has been described in several species, as obesity is associated with reductions in aerobic capacity, increased work effort (reduced efficiency of work), and altered energy metabolism [3].

Several of these elements are of great interest in the horse industry, as an increasing proportion of the horse population is overweight and/or obese, including some equestrian athletes. Many horses also suffer from insulin dysregulation (ID), which describes both insulin resistance and the resulting hyperinsulinemia. Horses may be described as having Equine Metabolic Syndrome (EMS) when they demonstrate criteria including ID, obesity and/or regional adiposity, hypertension, hypertriglyceridemia, and/or altered adipokine concentrations [4]. However, it should be noted that some horses and ponies may also suffer from insulin resistance without being overweight [5]. Laminitis is a debilitating hoof condition often requiring euthanasia, which may be triggered by multiple factors including sepsis, trauma, or endocrinopathic disorders, including EMS or pituitary pars intermedia dysfunction (PPID or Cushing’s disease). The term hyperinsulinemia-associated laminitis (HAL) has been coined to describe some of these cases, as elevated insulin concentrations in the blood can cause acute laminitis [6,7]. It is possible that exercise conditioning can mediate the severity of ID and facilitate weight loss, thus reducing the risk of laminitis, while also improving aspects of athletic work and mitigating OIDEC. Indeed, there are numerous reports documenting that exercise increases insulin sensitivity in horses, with or without changes in body composition or reductions in body fat.

## 2. Obesity and Insulin Resistance

In several species, adiposity is associated with the development of insulin resistance, with many individuals developing Type 2 diabetes. Insulin is secreted by the β-cells of the pancreas and its functions include the following: lowering blood glucose concentrations by stimulating glucose uptake by tissues and upregulating glucose oxidation and glycogen synthesis, inhibiting gluconeogenesis, contributing to fat storage by inhibiting lipolysis, and promoting triacylglycerol storage in adipose tissue. Insulin exerts its primary function on insulin-sensitive tissues by first binding to the insulin receptor (INSR) at the cell membrane [8]. The INSR has both α and β subunits, with insulin binding to the α subunits, which triggers a conformational change and causes the β subunits to activate a tyrosine kinase enzyme. This results in the autophosphorylation of the β subunit along with a series of other intracellular proteins known as insulin receptor substrates (IRSs). This signal transduction cascade results in the activation of phosphoinositol 3 kinase (PI3K); the recruitment and activation of PIP2, PIP3, and PDK-1; and then the activation of protein kinase B (PKB, also known as AKT). PKB/AKT functions to phosphorylate and deactivate proteins AS160 (also known as TBC1D4) and TBC1D1. This promotes the activation of the protein Rab-GTP, which causes the movement of the major glucose transporter, GLUT4, from intracellular vesicles to fuse with the cell membrane, thus allowing glucose to move through GLUT 4 into cells [9,10]. Once inside the cells, hexokinase acts to trap glucose inside by hydrolyzing ATP and converting glucose to glucose-6-phosphate. The activation of PKB/AKT also phosphorylates and, thus, inactivates glycogen synthase kinase 3 (GSK3). This results in the dephosphorylation and, therefore, activation of glycogen synthase to promote glucose storage [8]. Similar pathways also result in the activation of enzymes to promote the synthesis of fats and triglycerides. Insulin has further anabolic properties by facilitating the uptake of amino acids by tissues and promoting protein synthesis, as insulin and AKT also activate the mammalian target of rapamycin (mTOR) pathways [11,12].

It should be noted that there are several types of transporters that facilitate the diffusion of glucose, with most work examining the Class 1 transporters (GLUT 1–4). GLUT4 is the most prevalent transporter in the muscle, it is sensitive to insulin, and it has been widely studied. However, GLUT1 is a basal glucose transporter that is insulin-independent and allows glucose to move with its concentration gradient. GLUT2 and GLUT3 have important functions for glucose transport within the pancreas, kidney and liver (GLUT2), and the brain (GLUT3). GLUT 5, 7, 9, and 11 (Class 2 transporters) appear to move both glucose and fructose within several tissue types, including the small intestine and kidneys. GLUT 6, 8, 10, 12, and 13 are considered Class 3 transporters with expression in tissues including the brain, liver, and testes. The roles of several GLUT transporters are only now being discovered. Meanwhile, the sodium-dependent glucose-linked transporter (SGLT) is important for glucose transport in the gut and kidneys [13].

Insulin resistance (IR) describes a situation wherein insulin fails to exert its effects on tissues, which is most commonly documented by a decrease/failure of tissue glucose uptake in the presence of insulin. However, insulin resistance and its impaired signaling pathways also affects fat and protein metabolism.

It is recognized that adipose is not solely a tissue for energy storage. Adipose is an important endocrine organ that produces hormones called adipocytokines (e.g., adiponectin and leptin), which can contribute to energy balance. Adiponectin functions to regulate glucose and fat metabolisms, and concentrations are higher in lean individuals; and these concentrations decrease with weight gain and obesity. Studies administering adiponectin have reported an increase in glucose uptake through effects on the insulin receptor activity and the promotion of fatty acid oxidation [11,14]. With obesity, adiponectin is reduced, and these positive roles on glucose and fat metabolism are withdrawn. Leptin concentrations are lower in lean individuals and increase with adiposity, and leptin is involved with satiety and energy expenditure. Obesity contributes to hyperleptinemia, and eventually to leptin resistance. Excessive adipose tissue buildup (i.e., obesity) results in the over-production of numerous pro-inflammatory cytokines, particularly TNFα and IL6, as well as the adipocytokine resistin. The production of these compounds, in turn, contributes to chronic inflammation and can affect several other metabolic pathways. For example, increases in TNFα and IL6 lead to a decrease in insulin receptor activity, and TNFα further inhibits fatty acid and glucose metabolism [15,16]. In animal studies, resistin functions to inhibit insulin signaling and glucose uptake, thus causing insulin resistance. It should also be noted that excessive free fatty acids in circulation, which occur with obesity, may also override the ability of cells to metabolize them, resulting in lipotoxicity. Fat accumulation has been shown to impair the deactivation of AS160/TCB1D4, thus preventing the movement of GLUT4 [17]. Also, biologically active lipids, namely, diacylglycerols, ceramides, and long-chain acyl-CoA, appear to affect insulin signaling pathways. For example, ceramide inhibits the phosphorylation and activation of AKT [11]. Fat accumulation further results in the production of reactive oxygen species (ROS) at the mitochondria, which can also affect insulin signaling pathways [18]. The accumulation of adipose tissue, therefore, contributes towards the development of insulin resistance. Insulin resistance plays a key role in the dysfunction of pancreatic beta-cells and the development of type II diabetes mellitus (T2DM; [19]).

While the association between obesity, inflammation, and insulin resistance is strong, it should be noted that insulin resistance may develop in lean individuals as well. Some of these individuals may have more fat accumulating around organs (visceral fat) compared to subcutaneous fat and would, therefore, appear lean [20,21]. It is believed that a high intake of refined sugars, particularly fructose, triggers the production of inflammatory proteins and cortisol, which can contribute to insulin resistance [22]. Systemic inflammation plays a role in obesity-related insulin resistance but may also affect glucose and insulin metabolisms in individuals with other causes of inflammation [18].

Adiposity, along with its alterations in glucose metabolism, has consequences for athletic capacity [3,23,24]. While professional athletes are less likely to be overweight, amateur athletes may be [25,26]. Further, obese and overweight individuals who are prescribed exercise training as part of a fitness program may face greater challenges to a given workload [27]. Overweight individuals with and without T2DM have reduced glucose uptake and oxidation [28]. Further, obese skeletal muscle produces more lactate than lean muscle [29]. Obese humans have reduced aerobic capacity, as measured by VO_2max_ after accounting for fat mass [30]. Obese subjects are also more likely to end exercise due to musculoskeletal pain (vs. fatigue in lean but non-athletic individuals) [31]. Exercise capacity at several levels may be compromised with obesity, resulting in obesity-induced decline in exercise capacity (OIDEC).

## 3. Obesity and Insulin Resistance in Horses

Obesity in horses is characterized by excessive amounts of body fat accumulating in several parts of the body, including across the crest of the neck, along the shoulder and ribs, and across the tailhead area, as well as pronounced visceral fat. Body fat can be measured objectively in horses using methods including carcass evaluation, ultrasonography of subcutaneous fat, measurement of total body water using deuterium oxide, and bioelectric impedance [32,33,34]. Body fat may also be measured subjectively by evaluating the amount of subcutaneous fat. The most common method is the Henneke body condition scoring scale, which is a score of 1–9 used to describe the amount of fat coverage on a horse, wherein a horse with a score of 1 would be severely emaciated and a horse with a score of 9 would be grossly obese [35]. Most equine health professionals would agree that scores between 4 and 6 would be considered ideal, those horses with scores over 6 would be considered overweight, and those over with a score over 7 would be considered obese. Obesity may also be described in horses that have a “cresty neck” outlined by Carter et al. [36]. Other methods of describing regional adipose tissue have also been developed, such as the Equifat system [37]. Regardless of the system used, estimates of obesity in horses from around the world range from 22 to 62% of the horse population [32,38,39]. It is likely that overweight and obese horses tend to be more idle/leisure types of horses, though competitive equine athletes may also be overweight [40,41].

Obesity develops largely due to an imbalance between calorie intake and calorie expenditure. This could be because of simple overeating and a lack of exercise, as many horses of today are fed high-quality forages and grains, often with less exercise than horses were once used to. Some breeds of horses appear to be easy keepers, resulting in them being more prone to weight gain [42,43,44]. Evidence in humans suggests that genetics may contribute greatly to obesity (up to 40–70% of cases), supporting the notion that there may be a genetic link within some breeds [11]. Recent work also suggests that the equine microbiome may be involved in energy balance [45,46], where the microbes ferment fiber to produce volatile fatty acids, which can be a major source of energetic substrates for horses. For example, a horse that may be able to more fully ferment and digest a feed could generate more calories from it compared to an animal with lower fermentative activity and, thus, reduced digestibility of a feed.

Adiposity in horses also affects insulin metabolism. Insulin resistance (IR) has been documented in horses, though the condition is somewhat different from humans in that hyperinsulinemia alone can be very problematic, and the development of type 2 diabetes is rare. Therefore, the term insulin dysregulation (ID) more appropriately describes the collective abnormalities of insulin metabolism in horses [47]. The relationship between insulin resistance and hyperinsulinemia is described by Durham and others, where insulin resistance results in reduced glucose uptake, causing hyperglycemia. Hyperglycemia, in turn, stimulates the β-cells of the pancreas to release additional insulin. The potential decreased hepatic clearance of insulin also contributes to hyperinsulinemia. Meanwhile, hyperinsulinemia may additionally favor the further development of insulin resistance via a downregulation of signaling [4].

Insulin sensitivity (or insulin resistance) may be quantified using “gold standard” methods such as the euglycemic-hyperinsulinemic clamp (EHC), or through the minimal model analysis of a frequently sampled intravenous glucose tolerance test [47,48,49]. However, clinically, these practices are not often practical. Therefore, horse owners often rely on a single blood test to analyze glucose and insulin concentrations or proxies using these values [50], or a veterinarian can perform an oral glucose challenge test (OGT) [51]. Horses may be considered suspects for ID when resting (and no prior grain feeding) insulin concentrations are greater than 20–31 µU/mL (depending on the assay used), and ID is diagnosed if insulin concentrations are greater than 50–75 µU/mL (depending on assay). Confounding factors (such as stress or recent exercise) or differences in glucose absorption may affect these results. Further diagnostic descriptions are available (Equine Endocrinology Group, https://sites.tufts.edu/equineendogroup/. Assessed on 27 December 2023).

Insulin dysregulation develops in many horses due to obesity [44,51,52,53,54,55], likely via mechanisms described above. Diets high in starch and sugar have also been demonstrated to decrease insulin sensitivity, even in leaner animals [56,57]. Many horses exhibit resting (with or without hay/pasture or other feed) hyperinsulinemia, as well as exaggerated hyperinsulinemia following the feeding of a sugar- or starch-rich meal, or following the consumption of rich, lush pasture. Hyperinsulinemia is potentially problematic as it has been shown to directly cause laminitis [7], and there is an association between increased basal insulin concentrations and increased lameness (pain) due to laminitis [58]. Therefore, in many cases, while a true diagnosis of insulin resistance may be helpful, monitoring resting insulin concentrations can be a good management tool for owners and veterinarians to help identify horses that might be at risk of developing laminitis. Hyperinsulinemia has also been correlated to body condition score [39,59], with higher concentrations of insulin found in fatter horses. Indeed, hyperinsulinemia is closely associated with insulin resistance [48,50]. Again, the term Equine Metabolic Syndrome (EMS) is used to describe horses with insulin dysregulation, obesity, and/or localized fat deposits and other risk factors that increase the risk of laminitis [4,60,61,62]. It should be noted that not all obese horses have elements of ID, and some horses with IR and/or ID are not obese [63,64].

The link between obesity and insulin dysfunction is similar to the mechanisms presented above for humans. In horses, there is also an increased production of inflammatory cytokines with increasing adiposity, leading to low-grade inflammation [65,66,67,68]. In fact, adipocytokine production is correlated to body condition score and adiposity [39,59,66,69,70]. Obesity has also been shown to contribute to adipose tissue dysfunction in horses [53,71]. Oxidative stress is another component of both aging and obesity and may also contribute to alterations in insulin dynamics [18,69,72].

To this end, several studies have demonstrated that weight gain in horses results in reductions in insulin sensitivity. Further, a dietary energy source appears to influence insulin sensitivity in horses. Induced obesity with a high grain diet for 5 months resulted in ID (increased glucose and insulin concentrations, and higher area under a glucose curve following an OGT) after 90 days. After 150 days, there was significant fasting hyperinsulinemia as well as higher insulin concentrations following the OGT. Carter fed horses 200% of their energy requirements to achieve an increase of 2 body condition scores (avg BCS = 8) and reported that insulin sensitivity decreased by 71%, along with horses presenting with basal hyperinsulinemia and hyperleptinemia [55,73]. Also, d’Fonseca reported hyperinsulinemia along with higher insulin concentrations following an OGT in ponies fed to gain 27% of their body weight for 24 weeks but noted a consequentially more efficient glucose metabolism. Quinn and others documented that a body weight gain of almost 3 body condition scores (90 kg) only resulted in reduced insulin sensitivity (using the minimal model analysis) in horses fed a higher starch and sugar diet, but it did not affect those fed a diet higher in fat and fiber. Only after exercise was also restricted was there a negative impact on insulin sensitivity in the higher fat–fiber-fed horses [74]. Similarly, Pratt and others reported decreased insulin sensitivity in sedentary horses fed a diet high in starch and sugar compared to those fed a diet higher in fat and fiber, although with no changes in body weight or condition [56]. However, weight gain is not always associated with changes in glucose metabolism [75].

Excessive adiposity has its own potential health risks and can negatively impact exercise performance (see [32] for a review). Briefly, equine obesity has been found to cause an accumulation of adipose tissue around internal organs such as the kidney and heart [76,77]. Excess adipose tissue contributes to the weight of horses, and weight carriage has a clear impact on the effort required to exercise during both race-type events [78] and non-racing exercise [79,80,81]. Adiposity is also associated with movement asymmetry [82]. Overweight horses have a harder time dissipating heat, which could contribute to early fatigue [83]. Similar to other species, it is likely that adiposity contributes to mechanical load in joints and an earlier onset of arthritis. For example, in dogs, obesity is strongly associated with the development of osteoarthritis [84,85]. In horses, there are multiple factors that may affect the development of arthritis besides obesity (such as conformation or work-level). However, arthritis was recognized by horse owners as the most prevalent weight-related disorder in horses, in comparison to in ponies (where the most prevalent weight-related disorder was believed to be laminitis) [86]. Therefore, it is important for our equine partners to be leaner, both from an overall health standpoint and also in terms of being able to perform their best. Exercise conditioning has the potential to do both.

## 4. Exercise, Weight Loss, and Insulin Metabolism

Exercise is well recognized as being able to prevent, manage, and treat disease as a result of its anti-inflammatory effects, its ability to reduce body fat, and its effect on glucose and insulin metabolisms [87]. Both acute exercise and long-term exercise conditioning increase insulin sensitivity. A single bout of exercise increases insulin sensitivity for 12–72 h in a relationship that is relative to energy expenditure, such that higher-intensity exercise results in greater whole-body insulin sensitivity [88,89]. This partially occurs as a result of the contraction-mediated movement of GLUT4 to the cell membrane, independent of the action of insulin. This mechanism is primarily due to the role of adenosine monophosphate-activated protein kinase (AMPK). AMPK is considered a sensor of cellular energy status and is activated with stress and muscle contraction. With muscle contraction, there is an increased demand for ATP, which becomes depleted. This depletion shifts the AMP/ATP ratio, which activates the myokinase reaction (ADP + ADP > ATP + AMP) to produce additional ATP and AMP. Increased AMP activates AMPK [90]. AMPK deactivates AS 160 and TBC1D1, which results in the movement of GLUT4 to the cell membrane. While some believe that calcium released from the sarcoplasmic reticulum upon stimulation via the t-tubules also acts directly and indirectly (via calcium-regulated protein kinases) to increase AMPK [9], other studies suggest that calcium is not involved [91]. Regardless, Kjøbsted and others demonstrated that AMPK is activated through muscle contraction and appears to intensify the effects of insulin both at the muscle and the whole-body levels [90]. Increased blood flow to the muscle during exercise complements these effects by delivering additional glucose and any circulating insulin. Key glycolytic enzymes (ex. Phosphofructokinase) are activated through a low energy status and AMPK, resulting in increased glucose usage. Therefore, with exercise, both contraction-mediated independent glucose uptake and insulin-mediated glucose uptake are enhanced, along with overall glucose disposal, which, when combined with carbohydrate consumption, can result in rapid glycogen synthesis in most species [92]. This AMPK-mediated effect is generally recognized to last up 4 h after exercise, though a prolonged acute effect (PAE) of exercise may last up to 72 h after exercise [93]. Following exercise, AKT continues to facilitate GLUT4 translocation to the cell membrane via TBC1D4, which continues longer after exercise is complete [10], contributing to the post-exercise effects of exercise on insulin sensitivity.

Regular exercise conditioning may have additive effects of frequent singular-exercise bouts, as well as more general adaptations including a greater muscle mass and, therefore, a larger sink for glucose. Of note, GLUT4 mRNA expression and protein content increase with exercise training [94,95,96]. This appears to occur with both aerobic/endurance types of exercise training as well as resistance exercise. Moderate-intensity exercise training has also been shown to increase IRS activation [10]. Glycogen synthase activity also appears to be upregulated with exercise training, thus providing a greater sink for glucose, and exercise-trained athletes tend to have larger concentrations of glycogen in the muscle [96]. Exercise conditioning results in reduced vascular inflammation and improved capillarization to the muscles. The latter contributes to improved glucose delivery to the muscles [10]. Exercise conditioning appears to improve insulin sensitivity even without changes in adiposity [97], though these effects may be short-lived [98]. Regular exercise decreases inflammation and would alleviate the effect of ROS on insulin signaling [99,100].

A further effect of exercise on insulin sensitivity and glucose metabolism likely includes the contribution of exercise to weight loss and the alleviation of inflammation [87,101]. Exercise results in caloric expenditure, potentially shifting energy balance and resulting in weight loss. Reducing body fat would reduce the negative impact of adipose tissue as described above. Several studies have examined the effects of fat reduction surgery on insulin sensitivity, and most have reported some improvements in glucose metabolism [102,103]. However, insulin sensitivity is not always improved with weight loss alone; diet- and exercise-induced weight loss is important [104]. Thus, exercise and weight loss result in greater improvements in insulin sensitivity [98,101,105], particularly for those with some levels of insulin resistance. Weight loss also appears to ameliorate inflammation as well as other diseases including arthritis [87,106,107].

## 5. Exercise, Weight Loss, and Insulin Sensitivity in Horses

Due to the negative health consequences of obesity in horses, many studies have investigated the effects of either diet and/or exercise on weight loss and insulin dynamics in horses. Horses with active laminitis may not be able to exercise and, therefore, must rely primarily on dietary restriction to facilitate weight loss. Several studies have, therefore, investigated the effects of either dietary energy intake restriction or exercise (or both) on overall glucose metabolism.

A few studies have directly examined the impact of exercise on insulin sensitivity in horses using gold-standard methodology (Table 1). Powell and colleagues (2002) quantified insulin sensitivity using an EHC in lean (BCS 4.5–5) and obese (8.5–9) mares before exercise conditioning, and 24 h and 9 days following 7 days of light–moderate exercise in a round pen for 30 min (heart rate < 140 bpm). Without any changes in body weight or body condition, insulin sensitivity (as glucose-infusion rate during the EHC) increased 24 h after the last exercise bout but was back to basal levels 9 days after the cessation of exercise training [108]. Pratt and others reported that 7-week exercise conditioning in lean horses ameliorated the negative impacts of a high-starch and -sugar diet on insulin-sensitivity horses [56]. These horses demonstrated an increase in GLUT4 content and hexokinase activity [109]. It was also reported that 7 days of exercise (45 min at 55% VO_2max_) resulted in increased insulin sensitivity, GLUT-4 protein content, and glycogen synthase activity. Following an additional 5 days of inactivity, these increases were still present [110]. In contrast, Carter and others reported that 8 weeks of moderate-intensity exercise conditioning in overweight horses (BCS ≥ 7), which resulted in weight loss (4%) and fat mass loss (34%), was not sufficient to affect insulin dynamics as quantified by a minimal model analysis of a frequently sampled intravenous glucose tolerance test [111].

Studies have also aimed to determine the effects of exercise conditioning on whole-body glucose dynamics by measuring basal glucose and insulin concentrations and/or following a dynamic oral glucose challenge. Overweight (BCS = 8.4 ± 0.9), hyperinsulinemic ponies also showed improved insulin sensitivity through 6 weeks of exercise conditioning [112]. Turner and others put horses into one of three exercise protocols: turned out for self-exercise, light, or moderate exercise [113]. These horses were not overweight but were fed a high-concentrate diet. Insulin sensitivity was estimated using the reciprocal of the square root of insulin (RISQI [50]), and only forced exercise resulted in improvements. De Laat used a dynamic feeding system to increase daily distance traveled in horses. Compared to a stationary feeder, the dynamic feeding system increased distance traveled and weight loss (BCS 6.53 ± 0.94 to 5.38 ± 1.71) but was not sufficient to improve insulin sensitivity in all ponies studied [114]. Moore exercised obese horses for 4 weeks at a workload calculated to expend the equivalent of 15% of the horse’s DE requirements. Exercised horses showed improvements in insulin concentrations following an oral sugar test as well as reduced leptin concentrations [115].

Studies have investigated the combined effects of diet and exercise, in part to determine which may be more effective (Table 2). Bamford studied obese horses (BCS ≥ 7) that were placed on an energy-restricted diet compared to those who also underwent forced exercise 5 days per week. All horses lost similar amounts of weight and had reduced insulin and leptin concentrations along with increased adiponectin concentrations. However, the horses that also exercised had significantly improved insulin sensitivity, as assessed through a minimal model analysis of an intravenous glucose tolerance test [116]. Exercise, therefore, appears to have a greater impact on insulin sensitivity than dietary restriction. Similarly, Gordon and others fed horses a weight control feed plus controlled amounts of hay and compared those who exercised 3× per week, and they reported stronger improvements in horses that exercised [117]. This point was further proven in Moore’s study described above, where another treatment of horses underwent a 15% reduction in DE intake (compared to the 15% of DE calorie expenditure, thus both treatments effectively resulting in 85% of net estimated calorie requirements). While these diet-restricted horses also lost significant amounts of body weight and condition, these were greater in the exercised horses, and the diet-restricted group showed no improvements in insulin sensitivity [115]. Along these lines, Pagan reported that while some sport ponies were overweight, their insulin concentrations were not as elevated as might be expected, potentially due to the positive effects of exercise [40].

In many cases, the ability to exercise may be limited due to impaired health or the lack of ability of owners to implement an exercise program. Therefore, it is still important to employ dietary restrictions for weight loss in some horses, though energy and feed intake may need to be severely restricted. Weight loss to achieve 1% BW loss per week required the reduction of feed intake to 35% of energy requirements in some horses. However, this resulted in reduced insulin concentrations following an OGT and improved insulin sensitivity [118]. Reducing dry matter intake to 1% of body weight resulted in weight and fat loss, though insulin dynamics were not specifically measured in this study [119]. A body weight reduction plan (reduced energy intake) resulted in significant weight loss in ponies with diagnosed insulin resistance, leading to improved insulin sensitivity [70]. Weight loss in these ponies also resulted in reduced serum leptin concentrations and increased plasma adiponectin concentrations. Gill and coauthors worked directly with horse owners to develop individual dietary management strategies for their overweight horses, which included reducing/restricting pasture, switching from traditional concentrates to ration balancers, and weighing feed. Horses with owners that followed their assigned protocols lost weight and had ameliorated insulin concentrations [120]. Weight loss with dietary energy restriction (and, thus, feed intake restriction) may not be sufficient in some horses that appear to have weight loss resistance. In these cases, a very low feed intake (1% of body weight) was required for some horses to lose weight [121]. Therefore, some horses may need additional help losing weight. One study aimed to determine the effects of weight loss induced by thyroid hormone administration for 48 weeks. Frank and others administered levothyroxine to horses and reported significant weight loss without any observable negative consequences. The level of weight loss (~5–10% of body weight) was sufficient to result in almost 2-fold increases in insulin sensitivity [122]. These authors also reported that insulin sensitivity was negatively correlated with body weight.

Together, these studies document the important contributions of exercise and dietary restriction to facilitate weight loss, and, ultimately, to improve insulin sensitivity. Due to the large number of negative health and performance consequences of obesity and insulin resistance, it is important that horse owners manage their horses to promote leaner body types. While exercise appears to have superior benefits on overall glucose metabolism and insulin sensitivity, weight loss (through exercise, dietary restriction, or medication) is an important and achievable goal.

It should be noted that an interesting facet of glucose and insulin metabolism in horses is their relatively slow ability to replete muscle glycogen following a glycogen-depleting exercise bout compared to other species [123]. Horses do not appear to have the same post-exercise increase in insulin sensitivity and muscular glucose uptake as seen in other species, even in the face of hyperglycemia [124,125]. While GLUT4’s concentrations increase with exercise training in horses [110], its expression does not appear to increase significantly following glycogen-depleting exercise [126]. Of interest is the increase in the expression of other GLUT transporters (namely, GLUT3, GLUT6, and GLUT10) [126]. It is currently unknown how the expression of these transporters is influenced by exercise training. Further research is required to elucidate the functions of these transporters with respect to exercise, glucose metabolism, and glycogen synthesis.

## 6. Practical Tips to Use Exercise Conditioning to Counter the Effects of Obesity and Insulin Resistance

To summarize, below are some strategies that may be helpful to facilitate weight loss and promote healthier glucose metabolism. Veterinary supervision may be required in some cases.

- Exercise the horse to the highest level of intensity suited for his overall health.
  ⚬ Recall that exercise intensity is directly related to the benefits seen in glucose metabolism.
  ⚬ In horses with lameness issues, this might be limited to walking, but such horses may benefit from the use of equipment such as an underwater treadmill or swimming pool to decrease weight-bearing on limbs.
  ⚬ Exercise intensity may be increased even at low speeds by adding hill work and poles.
- Exercise the horse at least 3–6 days per week
  ⚬ This will help to ensure any acute effects of exercise may last the entire week to provide additive benefits to the longer-term benefits of exercise conditioning.
- Limit calorie intake while ensuring the rest of the diet is adequately balanced
  ⚬ Work closely with an equine nutritionist.
  ⚬ Reduce/remove energy-dense concentrates, particularly those rich in starch and sugar.
  ⚬ Avoid an uncontrolled grazing of pasture.
    ▪ Use a grazing muzzle if pasture is the only option.
  ⚬ Focus on forage—hay/preserved forages.
    ▪ Hay can be easily analyzed, soaked, and weighed to provide controlled and known nutrient intakes.
    ▪ Hay intake may need to be reduced to 1.5% of body weight and reduced further as needed (and under veterinary supervision) to achieve weight loss.
  ⚬ Try to feed as much forage as possible (by weight), while ensuring daily calorie intake is lower.
    ▪ Select hay with a lower caloric density (i.e., less than 1.7 mcal/kg DE; typically with more than 50% ADF).
    ▪ Soak hay for 30 min to reduce some sugars and calories (disperse the water after soaking).
    ▪ Use a slow-feed hay net to prolong feeding time.

## 7. Conclusions

Obesity is a global problem that has negative consequences on health and exercise performance. Adipose tissue promotes inflammation and insulin resistance, and it alters glucose metabolism, both at rest and during exercise. Adipose tissue accumulation also loads the skeletal system, thus increasing the amount of work for locomotion and potentially causing strain to the joints, tendons, and ligaments of the limbs. Therefore, there is an obesity-induced decline in exercise capacity in horses, as well as other species. Exercise promotes insulin sensitivity, upregulates glucose metabolism both at rest and during exercise, and also burns calories and, in most instances, reduces body fat. Exercise conditioning, therefore, has important benefits for the health of all horses, whether they are athletes or companions. Further research is required to elucidate the additional benefits of exercise conditioning in horses.

## Figures and Tables

**Table 1:** Effect of exercise on insulin sensitivity in horses.

Horse Type	Exercise	Diet	Test Used	Effect on Insulin Sensitivity	Other Findings	Reference
Lean and obese mixed breed mares	7 days of light–moderate exercise (~140 bpm, 30 min)	No diet treatment	EHC	Increased IS		[108]
Lean Standardbreds	7 weeks of moderate exercise	High SS	EHC	Increased IS in High SS similar to FF	Increased GLUT4	[56,109]
		High FF				
Lean Standardbreds	7 days of endurance exercise	No diet treatment	EHC	Increased IS by 2 times	Increased GLUT4	[110]
					Increased GS activity	
Overweight/obese Arabians	8 weeks of low- to moderate-intensity exercise	No diet treatment	MMA	No effect	Reduced fat 34%	[111]
Hyperinsulinemic ponies	6 weeks of moderate-intensity exercise	Controlled feed intake	OGTT	Decreased insulin response to glucose	Decreased body weight and condition	[112]

IS: Insulin sensitivity, EHC: euglycemic-hyperinsulinemic clamp, SS: diet high in starch and sugar, FF: diet high in fat and fiber, GS: glycogen synthase; MMA: minimal model analysis of a frequently sampled intravenous glucose tolerance test; OGTT: oral glucose tolerance test.

**Table 2:** Effect of dietary energy restriction and/or exercise on insulin sensitivity in horses.

Horse Type	Exercise	Diet	Test Used	Effect on Insulin Sensitivity	Other Findings	Reference
Overweight stock-type	4 weeks of exercise to expend 15% of DE requirements	No diet treatment	OST	Improved IS	Decreased leptin	[115]
					Decreased BW	
					Decreased BCS	
	No exercise	DE intake reduced by 15%		No change in IS	Decreased BW	
					Decreased BCS	
Obese horses and ponies	5 days of exercise	Energy restricted	MMA	No change in IS	Decreased BW	[116]
	25 min of walk/trot				Decreased leptin	
	No exercise	Energy restricted		Improved IS	Decreased BW	
					Decreased leptin	
Overweight QHs and TBs	12 weeks of moderate exercise (30 min up 1 h, ~65% HR max, 3× per week)	Weight control feed +1% BW hay	MMA	Improved AIRg	Decreased BW (~52 kg)	[117]
					Decreased BCS (~2)	
					Decreased leptin	
	No exercise			Improved AIRg	Decreased BW (25 kg)	
					Decreased BCS (~1)	

IS: insulin sensitivity, MMA: minimal model analysis of a frequently sampled intravenous glucose tolerance test; OST: oral sugar test; BW: body weight, BCS: body condition score, HR: heart rate.
